# Muscle MRI quantifies disease progression in amyotrophic lateral sclerosis

**DOI:** 10.1136/jnnp-2024-335571

**Published:** 2025-03-25

**Authors:** Uros Klickovic, Luca Zampedri, Nick Zafeiropoulos, Oliver J Ziff, Christopher DJ Sinclair, Stephen Wastling, Magdalena Dudziec, Jodie Allen, Karin Trimmel, Robin S Howard, Andrea Malaspina, Nikhil Sharma, Katie CL Sidle, Sachit Shah, Christian Nasel, Tarek A Yousry, Linda Greensmith, Jasper M Morrow, John S Thornton, Pietro Fratta

**Affiliations:** 1 MND Centre, UCL Queen Square Institute of Neurology, London, UK; 2 Department of Radiology, University Hospital Tulln, Tulln, Austria; 3 Neuroradiological Academic Unit, UCL Queen Square Institute of Neurology, London, UK; 4 Department of Neurology, Medical University of Vienna, Vienna, Austria; 5 Lysholm Department of Neuroradiology, National Hospital for Neurology and Neurosurgery, London, UK

**Keywords:** NEUROMUSCULAR, MRI, MUSCLE DISEASE, ALS

## Abstract

**Background and objectives:**

Quantitative and operator-independent biomarkers of disease progression are urgently needed in amyotrophic lateral sclerosis (ALS) research. We assess the potential of skeletal muscle MRI as a sensitive and reliable outcome measure for future ALS clinical trials.

**Methods:**

In this longitudinal cohort study, muscle MRI of head-neck, upper and lower limb regions, alongside clinical and functional assessments, were acquired at three time points over the individual maximum observation period (iMOP) of 1 year in 20 patients with ALS and 16 healthy controls. Quantitative MRI parameters cross-sectional area (CSA), volume (VOL), fat fraction, functional rest muscle area and water T2 (T_2m_) were correlated with changes in clinical disease severity (functional rating scales and myometry).

**Results:**

Among 20 patients with ALS, 17 completed follow-up. Progressive muscle atrophy (CSA, VOL) was observed at hand (rs=0.66), head-neck (partial η²=0.47) and lower-limb level (thighs: η²=0.56, calves: η²=0.54) over iMOP. MRI changes correlated with leg muscle strength (knee extension: r=0.77; plantar flexion: r=0.78), hand grip strength (r=0.71) and functional rating scales (r=0.68).

**Interpretation:**

Our findings demonstrate the effectiveness of muscle MRI as a sensitive neuroimaging biomarker of disease progression in ALS, highlighting its potential application in clinical trials.

**WHAT IS ALREADY KNOWN ON THIS TOPIC**

- Biomarkers of disease progression are urgently needed to minimise the size, duration and cost of clinical trials in amyotrophic lateral sclerosis (ALS) to allow a more effective investigation of promising therapeutic agents.

**WHAT THIS STUDY ADDS**

- We demonstrate that longitudinal quantitative MRI reliably detects progressive atrophy and decrease of functional rest muscle area, which correlate with progressive loss of muscle strength and global clinical disease progression measured with functional rating scales.

**HOW THIS STUDY MIGHT AFFECT RESEARCH, PRACTICE OR POLICY**

- Muscle MRI has the potential to be utilised as a biomarker for ALS disease progression and could contribute to reducing the size and duration of clinical trials facilitating future drug developments.

## Introduction

 In amyotrophic lateral sclerosis (ALS) research, sensitive and reliable measures of disease progression are urgently needed to reduce the size, cost and duration of clinical trials and facilitate the effective investigation of therapeutic agents.

Quantitative muscle MRI has previously been validated to detect increased muscle fat infiltration (fat fraction (FF))^1^ in slowly progressive neuromuscular disorders, including Duchenne muscular dystrophy,^2^ inclusion body myositis,^1^ as well as motor neuron diseases (MNDs) like spinal and bulbar muscular atrophy.^3^ In rapidly progressing neuromuscular diseases such as ALS, FF increases are less striking, while quantification of muscle atrophy^4^ may be of greater clinical relevance.

Here, we applied quantitative muscle MRI of the upper and lower limbs as well as the head-neck region in patients with ALS at three time points over 1 year to assess atrophy, intramuscular fat infiltration and oedema. MRI outcome measures were correlated with clinical disease severity over time to determine the full potential of muscle MRI as a neuroimaging biomarker for future clinical trials in ALS.

## Methods

All methods are provided in the supplementary material.

## Results

### Participant demographics and clinical findings

The study included 20 patients with ALS, of which 17 were available for follow-up, along with 16 healthy controls.^4^ Demographic and clinical features of study participants are detailed in online supplemental tables 2 and 3. Muscle strength and functional rating scales (ALSFRS-R and subscores) significantly declined over time in patients with ALS (online supplemental table 3).

### Muscle MRI detects widespread progressive atrophy and decrease in functional muscle area in ALS

**Figure 1:**
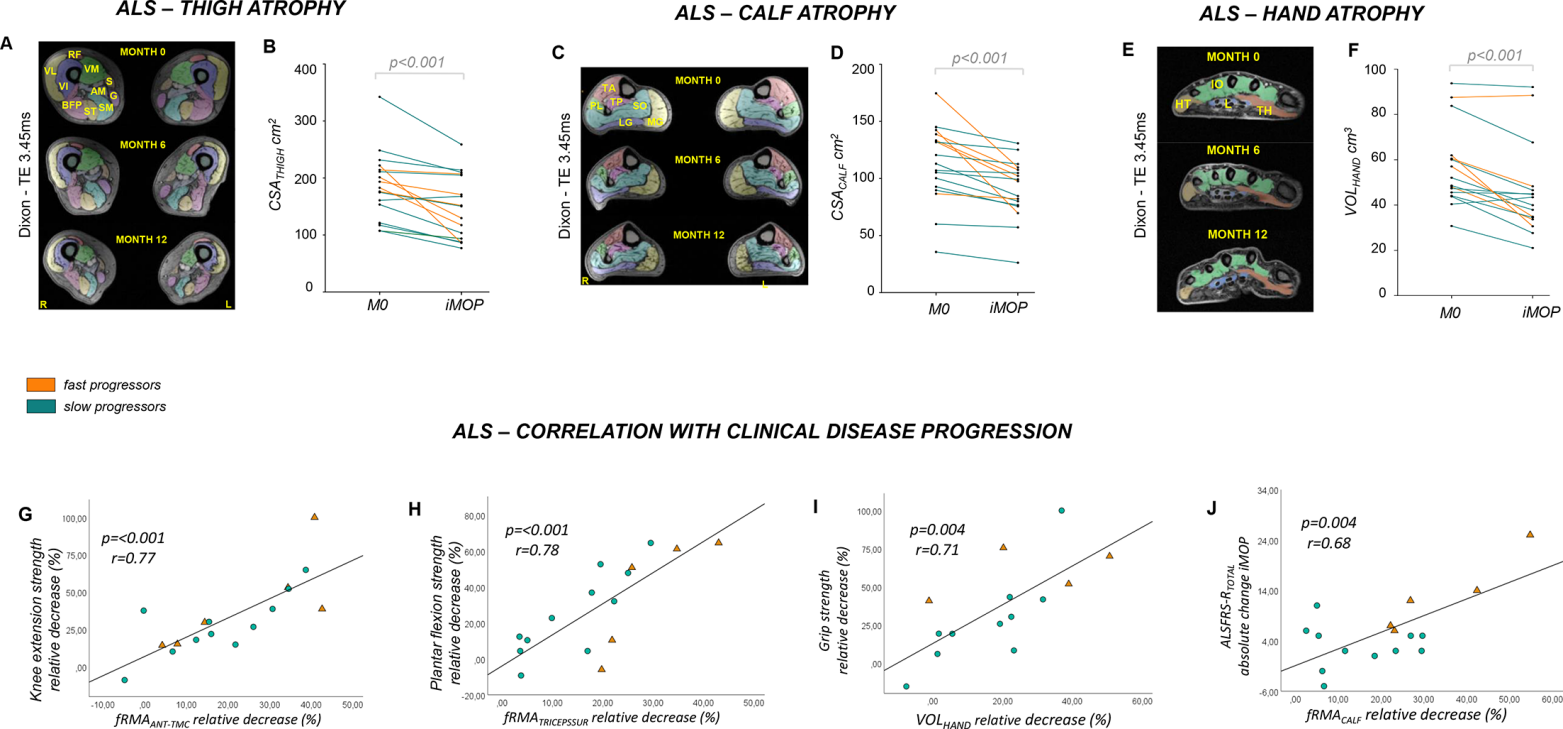
Muscle MRI detects progressive atrophy in lower limb and hand muscles in ALS and functional rest muscle area correlates with clinical disease progression. (A) Sample axial Dixon imaging (TE=3.45 ms) of thighs in patients with ALS at baseline (upper row), 6 months (middle row) and 12 months (lower row). (B) Mean values of overall cross-sectional area at thigh level (CSA_*THIGH*_) in patients with ALS (fast progressors: orange; slow progressors: green) at baseline (M0) and iMOP. CSA_*THIGH*_ significantly decreased over the iMOP (T(1,16)=4.51, p<0.001). (C) Sample axial Dixon imaging (TE=3.45 ms) of calves in a patient with ALS at baseline (upper row), 6 months (middle row) and 12 months (lower row). (D) Mean values of overall CSA at calf level (CSA_*CALF*_) in patients with ALS (fast progressors: orange; slow progressors: green) at baseline (M0) and iMOP. CSA_*CALF*_ significantly decreased over iMOP (T(1,16)=4.30, p<0.001). (E) Sample axial Dixon imaging (TE=3.45 ms) of the dominant hand of a patient with ALS at baseline (upper row), 6 months (middle row) and 12 months (lower row). (F) Mean values of hand volume (VOL_*HAND*_) in patients with ALS (fast progressors: orange; slow progressors: green) over time. VOL_*HAND*_ significantly declined over the iMOP (test statistic=–122, p<0.001). (G) At thigh level, a correlation of the relative decrease of fRMA_*ANT-TMC*_ with the relative decrease of knee extension strength over the iMOP (r=0.77, p<0.001) was observed. (H) At calf level, the relative decrease of fRMA_*TRICEPSSUR*_ significantly correlated with the corresponding relative decrease of plantar flexion strength over iMOP (r=0.78, p<0.001). (I) At hand level, significant correlations were observed between the relative decrease in VOL_*HAND*_ and the relative decrease of the grip strength over iMOP (r=0.71, p=0.004). (J) The relative change of fRMA*_CALF_* showed a significant correlation with the absolute change of ALSFRS-R_TOTAL_ score over the iMOP (r=0.68, p=0.004). ALS, amyotrophic lateral sclerosis; ALSFRS-R, ALS functional rating scale—revised; ALSFRS-R_*TOTAL*_, ALS functional rating scale total score; ALSFRS-R_*HAND-SS*_, ALS functional rating scale hand subscale score; CSA, cross-sectional area; CSA_*THIGH*_, overall cross-sectional area of thigh muscles; CSA_*CALF*_, overall cross-sectional area of calf muscles; fRMA_*ANT-TMC*_, muscle-specific functional remaining muscle area of anterior thigh muscle compartment; fRMA_*TRICEPSSUR*_, muscle-specific functional remaining muscle area of triceps surae muscles; iMOP, individual maximum observation period; M0, baseline; M6, 6-month follow-up; M12, 12-month follow-up; VOL_*HAND*_, hand volume. Muscle abbreviations: AM, adductor magnus; BFP, biceps femoris posterior; G, gracilis; HT, hypothenar; IO, interossei; L, lumbricales; LG, lateral gastrocnemius; MG, medial gastrocnemius; PL, peroneus longus; RF, rectus femoris; S, sartorius; SM, semimembranosus; SO, soleus; ST, semitendinosus; TA, tibialis anterior; TH, thenar; TP, tibialis posterior: VL, vastus lateralis; VI, vastus intermedius; VM, vastus medialis.

In patients with ALS, the cross-sectional area at thigh level (CSA_*THIGH*_) significantly decreased over the iMOP (partial η²=0.56; T(1,16)=4.51, p<0.001; table 1, figure 1). At calf level, CSA_*CALF*_ also decreased significantly (partial η²=0.54; T(1,16)=4.30, p<0.001; table 1, figure 1). Overall hand muscle volume (VOL_*HAND*_) significantly declined over the iMOP (rs=0.66; test statistic=–122, p<0.001); table 1, figure 1). Exploratory analyses of hand muscle subcompartments are provided in online supplemental figure 1. At head-neck level, the volume of the bilateral pterygoideus lateralis muscle (VOL_*PL*_) declined significantly over the iMOP (partial η²=0.47; T(1,15)=3.67, p=0.002; table 1, online supplemental figure 2).

**Table 1:** Quantitative MRI parameters in patients with ALS and controls at baseline and follow-up (maximum observation period).

	ALS				Controls			
	**Baseline**	**Follow-up**	**P value**	**95% CI**	**Baseline**	**Follow-up**	**P value**	**95% CI**
**Atrophy**								
CSA*_CALF_* (cm^2^)	112.81±33.17	91.24±26.66	**<0.001**	−32.22 to −0.93	146.64±36.36	143.40±32.61	0.13	−2.63 to 9.11
CSA*_THIGH_* (cm^2^)	183.33±59.24	148.08±56.23	**<0.001**	−56.02 to −20.21	235.98±59.12	238.59±67.19	0.48	−10.9 to 21.87
VOL*_HAND_* (cm^3^)	57.04 (16.39)	41.69 (13.98)	**<0.001**	−16.13 to −1.64	73.28±16.43	70.56±17.88	0.37	−3.30 to 1.30
VOL*_T_* (cm^3^)	18.33 (6.59)	13.57 (8.17)	**<0.001**	−6.73 to −0.37	26.77±6.60	26.86±6.43	0.85	−0.84 to 1.01
VOL*_HT_* (cm^3^)	8.30 (2.65)	6.45 (2.66)	**0.002**	−2.6 to −0.62	10.26±2.91	10.07±2.81	0.86	−0.53 to 0.63
VOL*_IO_* (cm^3^)	25.22 (8.67)	18.74 (8.28)	**<0.001**	−6.18 to −0.97	29.60±6.57	29.01±6.36	0.83	−1.35 to 1.09
VOL*_PL_* (cm^3^)	14.24±3.92	13.28±3.70	**0.002**	−1.51 to −0.40	15.62±4.84	15.43±4.91	0.38	−0.26 to 0.63
**Fat infiltration**								
FF*_CALF_* (%)	3.34 (2.07)	4.89 (7.77)	**0.002**	0.08 to 4.43	1.92 (1.08)	1.92 (0.84)	0.39	−0.37 to 0.16
FF*_THIGH_* (%)	2.79 (1.13)	2.95 2.24)	0.04	−0.08 to 1.2	1.79 (0.98)	1.70 (1.78)	0.49	−0.14 to 0.33
FF*_HAND_* (%)	2.22 (3.47)	5.83 (4.81)	**<0.001**	1.01 to 6.08	2.13 (1.93)	1.75 (1.38)	0.45	−1.78 to 1.52
FF*_TONGUE_* (%)	8.50±3.42	11.04±6.89	0.25	−1.93 to 6.82	8.51±5.98	9.44±5.08	0.59	−4.57 to 2.70
fRMA*_CALF_* (cm^2^)	108.48±32.67	85.40±27.31	**<0.001**	−35.69 to −12.46	138.82±32.73	136.56±29.40	0.45	−4.04 to 8.57
fRMA*_THIGH_* (cm^2^)	178.12±57.59	143.02±55.82	**<0.001**	−55.51 to −20.32	231.01±58.18	233.26±65.71	0.51	−10.91 to 20.94
fRMA*_TONGUE_* (cm^2^)	25.94±4.97	24.29±5.54	0.52	−4.49 to 2.38	27.11±4.52	25.48±3.88	0.21	−1.05 to 4.30
**T**_**2m**_								
T_2m*CALF*_ (ms)	34.61±3.85	36.87±5.48	0.01	−3.90 to −0.63	29.67±0.81	29.50±0.94	0.28	−0.16 to 0.50
T_2m*THIGH*_ (ms)	31.43±1.34	32.30±2.01	0.04	−1.67 to −0.07	29.63±1.00	29.54±0.83	0.60	−0.27 to 0.45

Data are presented as mean±SD or median (IQR) according to data distribution.

Significant p-values (Bonferroni-corrected) are highlighted in bold.

ALS, amyotrophic lateral sclerosis; CSA, cross-sectional area; FF, fat fraction; fRMA, functional rest muscle area; T_2m_, water T2; VOL, volume.

MRI data responsiveness was assessed using standardised response mean (SRM; mean change divided by the change SD) and highest responsiveness of atrophy measurements was observed for VOL*_HAND_* (SRM 1.17), followed by CSA*_THIGH_* (SRM 1.09) and CSA*_CALF_* (SRM 1.08; online supplemental table 4).

Functional rest muscle area (fRMA) significantly decreased both at thigh (fRMA*_THIGH_*; partial η²=0.57; T(1,16)=4.57, p<0.001; table 1) and calf level (fRMA*_CALF_*; partial η²=0.57; T(1,15)=4.42, p<0.001; table 1). Highest responsiveness was observed for fRMA*_THIGH_* (SRM 1.11) and fRMA*_CALF_* (SRM 1.10; online supplemental table 4). Details on FF analyses are provided in table 1 and online supplemental figure 3.

T_2m_ was used to assess the extent of muscle oedema^5^ and showed a trend to decrease over time at thigh and calf level over the iMOP (table 1, online supplemental figure 3, Bonferroni-corrected p>0.003). No significant changes in MRI parameters were observed over time in controls at lower limb, hand or head-neck level (table 1). Exploratory analyses investigating only the 6-month interval from baseline in all patients with ALS confirmed the changes detected over the iMOP, with an additional increase of T_2m*CALF*_ (online supplemental table 5).

### Progressive atrophy and decrease of functional muscle area correlate with disease severity

To assess whether muscle MRI changes are linked to functional measures and can be used to monitor ALS, we assessed their correlation with muscle strength measures. The relative decrease of fRMA*_ANT-TMC_* of the anterior thigh muscle compartment significantly correlated with the relative decrease of knee extension strength over time (r=0.77, p<0.001; online supplemental file 1, figure 1). In the calf, the relative decrease of fRMA*_TRICEPSSUR_* significantly correlated with the relative decrease of plantar flexion strength over the iMOP (r=0.78, p<0.001; online supplemental table 6, figure 1).

We then assessed whether MRI findings correlated with functional rating scales and observed a significant correlation between the overall relative decrease of fRMA and decrease of the ALSFRS*_TOTAL_* score over the iMOP at calf level (r=0.68, p=0.004; online supplemental table 6, figure 1), while correlations at thigh level did not withstand Bonferroni adjustment (p>0.004, online supplemental table 6, figure 1). Correlations with ALSFRS lower limb subscale (ALSFRS*_LL-SS_*) and fRMA of thighs and calves remained non-significant (online supplemental table 6).

At hand level, the relative decrease of VOL*_HAND_* significantly correlated with the corresponding relative decrease of grip strength over time (r=0.71, p=0.004, online supplemental table 6, figure 1). The correlations between VOL*_HAND_* and ALSFRS*_HAND-SS_* revealed a trend for correlation (Bonferroni-corrected p>0.004, online supplemental table 6, figure 1). At head-neck level, longitudinal fRMA changes correlated with changes in the ALSFRS bulbar subscale (ALSFRS*_BULBAR-SS_*) over time (supplementary material). Additional exploratory analyses were performed to compare correlations in fast versus slow progressors, as well as probe the relation of MRI changes with the respiratory subscale of the ALSFRS-R. Details of these analyses are provided in the supplementary material.

## Discussion

To our knowledge, this is the first study to conduct a comprehensive longitudinal analysis of MRI parameters muscle atrophy, fRMA and oedema across limb, hand and bulbar muscles in ALS, and to correlate these changes with functional and clinical measures. Furthermore, this represents the largest muscle MRI-based study to date investigating progressive quantifiable muscle atrophy in ALS, providing a robust foundation for future biomarker development.

Over a maximum observation period of 1 year, we detected progressive, quantifiable muscle wasting in all investigated regions, which was most marked in the dominant hand (SRM −1.17) and leg muscles (SRM thigh: −1.09, SRM calf: −1.08). Exploratory analyses at 6-month follow-up confirmed quantitative MRI parameter changes already at this time point. Muscle FF primarily increased in calf rather than thigh muscles, which is supported by recently published longitudinal data on muscle FF in ALS.^6^ Longitudinal MRI changes correlated strongly with clinical muscle strength measurements. Correlations with ALSFRS-R and its subscores were less pronounced compared with myometry results. This is in line with previous observations that have questioned whether the ALSFRS-R is sufficiently sensitive to detect meaningful changes over time, given its ordinal, non-linear weighting, along with observations that disease severity may be underestimated on ALSFRS-R compared with objective examinations.^7^ Exploratory correlations with respiratory function (ALSFRS-R*_RESP_*) emphasise the need to explore respiratory muscles in detail in future studies and also consider the potential impact of respiratory function on muscle atrophy in ALS.

T_2m_ demonstrated a trend towards an increase over time in the lower limbs, predominantly at calf level. This aligns with previous studies suggesting that T2 signal changes in ALS reflect the accumulation of extracellular fluid caused by active muscle denervation in ALS.^8^ These changes are linked to functional impairment, as evidenced by the loss of muscle strength and motor unit number, with the calf muscles often being among the earliest and most prominently affected in ALS.^6 9^

Compared with whole-body MRI applications,^9^ our volumetric imaging protocol of distinct anatomical regions provides comparable features of an imaging biomarker of disease progression in all patients with ALS, with the advantage of shorter scanning times.

Due to the natural course of this progressive and fatal disease, data from approximately half of the initially enrolled patients were not available at the end of follow-up. Respiratory deficits make undergoing a conventional MRI challenging for patients, and shorter scanning protocols and upright positioning should be implemented for advanced disease stages.

Future studies in larger cohorts with shorter scanning intervals, combined with detailed clinical assessments, disease staging systems (such as King’s clinical staging)^10^ and comparison to other outcome measures such as forced vital capacity^11^ or biofluid biomarkers^12^ are warranted to validate and expand on our findings. This will help confirm our findings and determine the clinical significance of progressive muscle MRI changes in MND, particularly in terms of their utility for clinical monitoring and therapeutic intervention.

Patient survival is frequently used as an endpoint in clinical trials of ALS,^13^ but objective markers of disease progression may reduce the size and duration of future trials. We demonstrate that MRI-quantified atrophy of the hand and lower limbs showed the highest responsiveness to detect disease progression, further validated by strong correlation with patient muscle strength and functional scores. Our findings might have a substantial impact on the design of future clinical trials in ALS, potentially allowing a reduced number of participants needed to detect effects, with the ultimate goal of facilitating the development and approval of therapeutic agents in ALS. There is an urgent need for the development of new drugs in ALS, and our longitudinal study contributes to a small but fast-rising field of muscle MRI in MND and supports its integration in future interventional trials.

## Supplementary material

10.1136/jnnp-2024-335571 online supplemental file 1

## Data Availability

Data supporting the findings of this study are controlled by the respective centres and are not publicly available. Request to access the raw data should be forwarded to data controllers via the corresponding author. Written requests for access to the derived data will be considered by the corresponding author and a decision made about the appropriateness of the use of the data.
