# Supplementary material for: Muscle MRI quantifies disease progression in amyotrophic lateral sclerosis
Source: J Neurol Neurosurg Psychiatry. 2025 Mar 25;96(9):e335571. doi: 10.1136/jnnp-2024-335571 (PMC12418527; doi:10.1136/jnnp-2024-335571)
Supplement: online supplemental file 1 [file jnnp-96-9-s001.docx]

**Supplementary Material**

# METHODS

## Study Design and patient recruitment

We performed a prospective longitudinal observational study in 20 consecutive male and female patients with ALS who were recruited from the motor neuron disease clinic at the National Hospital for Neurology and Neurosurgery, Queen Square, London, UK between 2015 and 2017. All patients had a history of at least clinically probable disease according to revised El Escorial criteria^1^ (12 probable, 8 definitive). Out of 20 ALS patients, 17 patients were available for 6-month follow-up and 10 patients for 12-month follow-up, which represented the individual maximum observation period (iMOP). The cohort was furthermore stratified according to the monthly decrease in ALS Functional Rating Scale - Revised (ALSFRS-R) score into slow (≤0.5 point per month, n=11), and fast (≥ 1.0 point per month, n=6) disease progressors. Sixteen healthy controls with matched demographic characteristics formed the control group, of which 15 were available for follow-up. Exclusion criteria were concomitant neuromuscular diseases and MRI safety-related contraindications. ALS patients received clinical assessments and muscle MRI of the hand, thighs, calves, and head-neck region at baseline as well as 6-month and 12-month follow-up. Healthy controls were examined at baseline and at 12-month follow-up (iMOP). Cross-sectional data of baseline measurements in ALS patients and healthy controls, combined with data from other study participants, have been reported previously^2^. **Supplementary Table 1** details the numbers of participants at each timepoint and parameters recorded, along with reasons for exclusion.

## Standard Protocol Approvals, Registrations, and Patient Consents

The UCL Research Ethics Committee gave ethical approval for this work (11/LO/1425) and written informed consent was obtained from all participants.

## Clinical and functional testing

Patients and healthy controls were functionally rated using the ALS Functional Rating Scale-Revised (ALSFRS-R)^3^. The ALSFRS-R consists of 12 questions that each have a score from 0-4, where 4 indicates normal function and 0 indicates no function^3^. All study participants underwent detailed upper and lower limb myometry on a wireless handheld microFET®2 handheld dynamometer (Hoggan Scientific; UT, USA) for isometric assessment of jaw opening, wrist extension and flexion, finger extension and flexion, thumb abduction and adduction, knee extension and flexion, ankle extension and flexion. Handgrip strength and lateral pinch (between the radial side of the index finger and the thumb) were assessed with a Martin Vigorimeter (GP Supplies; London, UK)^4^. Except for jaw opening, all measurements were performed bilaterally. Iowa-Oral-Pressure-Instrument (IOPI) measures were carried out to asses the tongue pressure and bilateral peri-oral pressure^5^. IOPI measures tongue strength by using an inflatable tongue bulb with a pressure sensor. All asessments consisted of 3 attempts of 3-5 seconds of which the best attempt was selected. IOPI could not be acquired in four patients at baseline due to technical issues. All patients and controls additionally received detailed clinical assessments including medical history as well as clinical and neurological examinations.

## MR Imaging

Images of the participants' thighs, calves, hand and head-neck region were acquired at on a 3 Tesla Skyra MR system (Siemens Healthineers, Erlangen, Germany). Quantitative fat-fraction maps of the lower limbs, hand and head-neck were produced from the 3-point Dixon images (Thighs and calves: 2D gradient echo, nine 6 mm slices, TR = 102 ms, TE = 3.45/4.60/5.75 ms, flip-angle 10^◦^, NSA = 4, FOV = 420 x 180 mm, voxel size 1.3 x 1.3 x 6 mm^3^; Hand: 2D gradient echo, nine 6 mm slices, TR = 102 ms, TE = 3.45/4.60/5.75 ms, flip-angle 10^◦^, NSA = 4, FOV = 180 x 90 mm, voxel size 0.56 x 0.56 x 6 mm^3^; Head-neck: 2D gradient echo, eleven 10 mm slices, TR = 125 ms, TE = 3.45/4.60/5.75 ms, flip-angle 10^◦^, NSA = 3, FOV = 235 x 180 mm, voxel size 0.56 x 0.56 x 10 mm^3^) as described in detail previously^2^. T2 relaxometry data were acquired with a multi-echo-spin-echo sequence (Thighs and calves: Nine 6 mm slices, TR = 3500 ms, 22 echoes TE = 10 to 220 ms in 10 ms steps, NSA = 1 FOV = 420 x 180 mm, voxel size 1.3 x 1.3 x 6 mm^3^).

Total scan time for all sequences at multiple anatomical regions, including patient re-positioning, was about 60 minutes.

## MRI Data Analysis

A single experienced radiologist (U.K.) blinded to study groups outlined the muscles in the hand, thighs, calves and head-neck region using ITK-SNAP^6^. These regions were used to calculate quantitative MRI measures fat fraction (FF), cross-sectional area (CSA), and functional remaining muscle area (fRMA) for each muscle and muscle compartment group as well as a weighted overall mean muscle FF, described in detail previously^2^.

We calculated the overall mean fat fraction (FF) for each anatomical region of interest (ROI) by taking a weighted average across both sides of the body. The formula used for this calculation is as follows:

$$\mathrm{FF}=\frac{\sum_{i=1}^{2} \sum_{j=1}^{m} n_{ij}.FF_{ij}}{\sum_{i=1}^{2} \sum_{j=1}^{m} n_{ij}}$$

where *i =*1 for the left side, and *i* = 2 for the right. *m* represents the total number of muscle regions analysed at each anatomical level. *n_ij_* is the number of voxels in muscle *j* on side *i*.

To further characterise muscle quality, we calculated the functional rest muscle area (fRMA), which reflects the area of muscle tissue not replaced by fat. This was calculated using the following equation:

$$\mathrm{fRMA}=\frac{CSA x \left( 100-FF \right)}{100}$$

where CSA is the cross-sectional area of the muscle, and FF represents fat fraction. This metric allows to evaluate changes in muscle tissue over time and provides insights into the progressive nature of muscle atrophy in ALS.

Water T2 (T_2m_) estimation: A multi-component, slice profile-corrected EPG model [s(TE) = (1 - ffa) · sEPG(B1f, T_2m_, α, σN, TE) + ffa · [ 0.33 · sEPG(B1f, T2=40ms, α, σN, TE) + 0.67 · sEPG(B1f, T2=198ms, α, σN, TE)] was fitted pixel-wise to the data using maximum likelihood estimation in MATLAB, to estimate T_2m_ and an apparent fat fraction ffa. The 2-component fat-signal model parameters were estimated a priori from 4 subcutaneous fat ROIs in 8 representative subjects. Muscles were manually segmented on single slices at thigh and calf level. Mean T_2m_ and ffa were calculated for the entire musculature cross section at each level. Additionally, a manual slice-by-slice segmentation was performed on 2D gradient echo Dixon sequences (TE 3.45ms) to calculate the muscle volume of the dominant hand.

## Statistical Analysis

Statistical analyses were performed with GraphPad Prism version 9.1.2 with a p-value threshold of 0.05 with Bonferroni correction for multiple comparisons. As appropriate to the distribution of data, measures are reported as mean ± SD or median ± interquartile range (IQR). For between-group comparisons, 2-sample t-tests or Mann-Whitney-U-tests were applied, and for within-group comparisons (change over time in individual maximum observation period), paired t-tests or Wilcoxon signed-rank tests were applied as appropriate. Missing data were excluded from analyses. Correlations of MRI data with clinical measures were investigated with Spearman (rho) or Pearson coefficients as appropriate for the distribution of data. Differences between correlations were assessed with Fisher`s r to z transformation. Effect sizes are reported as partial η² respectively Spearman's Rank Correlation Coefficient. MRI data responsiveness was assessed using standardized response mean (SRM; mean change divided by the change SD).

**Supplementary Table 1.** Numbers of participants at each timepoint, parameters excluded from analyses in patients and controls and reasons for exclusion.

| **Parameter** | **Baseline** | | | | **6-month follow up** | | **12-month follow up** | | | | **iMOP** | |
| --- | --- | --- | --- | --- | --- | --- | --- | --- | --- | --- | --- | --- |
|  | **ALS (n=20)** | | **Controls (n=16)** | | **ALS (n=17)** | | **ALS (n=10)** | | **Controls (n=15)** | | **ALS (n=17)** | **Controls (n=15)** |
|  | **available** | **excluded (reason)** | **available** | **excluded (reason)** | **available** | **excluded (reason)** | **available** | **excluded (reason)** | **available** | **excluded (reason)** | **n** | **n** |
| **VOL*_HAND_*** | 20 | 0 | 16 | 0 | 16 | 1 (incomplete scan) | 9 | 1 (incomplete scan) | 14 | 1 poor image quality) | 16 | 14 |
| **VOL*_PL_*** | 16 | 4 (poor image quality; all subsequent loss of follow-up) | 15 | 1 (incomplete scan; subsequent loss of follow-up) | 16 | 1 (poor image quality) | 9 | 1 (poor image quality) | 15 | 0 | 16 | 15 |
| **FF*_HAND_*** | 17 | 3 (image processing failure) | 16 | 0 | 14 | 2 (1 incomplete scan, 1 image processing failure) | 9 | 1 (loss of follow up) | 11 | 4 (2 image processing failure, 2 incomplete scan) | 13 | 11 |
| **FF*_TONGUE_*** | 16 | 4 (poor image quality; all subsequent loss of follow-up) | 14 | 2 (1 image processing failure, 1 poor image quality with subsequent loss of follow-up) | 13 | 4 (2 poor image quality, 2 image processing failure) | 7 | 3 (2 poor image quality; 1 incomplete scan) | 13 | 2 (image processing failure) | 13 | 13 |
| **T_2m_*_THIGH_*** | 16 | 4 (poor image quality, 2 subsequent loss of follow-up | 15 | 1 (image processing failure) | 15 | 2 (poor image quality | 7 | 3 (image processing failure) | 14 | 1 (image processing failure) | 15 | 14 |
| **T_2m_*_CALF_*** | 19 | 1 (image processing failure, subsequent loss of follow-up) | 16 | 1 (image processing failure, subsequent loss of follow-up) | 16 | 1 (poor image quality | 10 | 0 | 15 | 0 | 16 | 15 |
| **Hand myometry** | 16 | 4 (unable to perform, 3 subsequent loss of follow-up) | 16 | 0 | 15 | 2 (incomplete examination, subsequent loss of follow-up) | 10 | 0 | 15 | 0 | 15 | 15 |

Note: FF = fat fraction; iMOP = individual maximum observation period; PL = pterygoideus lateralis muscle; VOL = volume; T_2m_ = water T2

**Supplementary Table 2.** Demographic data in ALS patients and controls. Data are presented as mean±SD or median (IQR) according to data distribution.

|  | **ALS (n=20)** |  | **Controls (n=16)** | **p** |
| --- | --- | --- | --- | --- |
| Sex (male/female) | 14/6 |  | 11/5 | 0.61 |
| Handedness (right/left) | 19/1 |  | 15/1 | 0.70 |
| Age (years) | 60.5 (24.5) |  | 62.0 (24.5) | 0.72 |
| BMI | 22.9±3.1 |  | 26.4±4.0 | **0.01** |
| Disease duration (years) | 2.0 (1.4) |  | n.a. | n.a. |
| Bulbar involvement (yes/no) | 8/12 |  | n.a. | n.a. |
| Riluzole therapy (yes/no) | 15/5 |  | n.a. | n.a. |
| Bulbar onset/Limb onset | 2/18 |  | n.a. | n.a. |

**Supplementary Table 3.** Functional rating scales and myometry results in ALS patients at baseline and follow-up (individual maximum observation period, iMOP). Data are presented as mean±SD or median (IQR) according to data distribution.

|  | **Baseline** | **Follow-up** | **p** |
| --- | --- | --- | --- |
| ALSFRS-R_TOTAL_ | 41.0 (2.0) | 35.0 (10.0) | **<0.001** |
| ALSFRS-R_LL-SS_ | 6.0 (3.5) | 4.0 (3.0) | **0.01** |
| ALSFRS-R_BULBAR-SS_ | 12.0 (2.5) | 11.0 (4.5) | **0.01** |
| ALSFRS-R_HAND-SS_ | 6.0 (1.5) | 4.0 (4.0) | **0.002** |
| Knee extension (Nm) | 59.5±18.3 | 36.4±17.4 | **<0.001** |
| Knee flexion (Nm) | 59.8±22.7 | 32.7±18.7 | **<0.001** |
| Dorsal extension (Nm) | 36.4±21.5 | 23.3±19.7 | **<0.001** |
| Plantar flexion (Nm) | 35.7±14.4 | 24.2±15.9 | **0.001** |
| Hand grip (Nm) | 124.7±45.1 | 76.9±47.9 | **<0.001** |
| Hand pinch (Nm) | 46.5±37.7 | 28.4±31.4 | **<0.001** |
| Jaw opening (Nm) | 17.9±5.1 | 12.1±1.9 | **0.001** |
| IOPI (kPa) | 40.4±14.2 | 35.5±15.8 | 0.13 |

**Exploratory longitudinal analysis of progressive hand volume (VOL*_HAND_*) changes in hand muscle subcompartments**

Subanalyses of the hand included single muscle volumes of the thenar (VOL*_T_*), hypothenar (VOL*_HT_*) and interossei muscles (VOL*_IO_*); with a significant decrease of muscle volume in each subgroup over iMOP (VOL*_T_*: rs=0.59, test statistic=-120, p<0.001; VOL*_HT_*: rs=0.59, test statistic=-114, p=0.002; VOL*_IO_*: rs=0.59, test statistic=-124, p<0.001, 95%CI= -6180 - -970). To investigate differences in wasting patterns between thenar and hypothenar muscles, we performed an exploratory two-way repeated measures ANOVA, which revealed a significant interaction effect of muscle compartment over time (F(1,30)=6,15; p=0.02). This indicates a more marked decline in the thenar compartment compared to hypothenar compartment, aligning with the concept of a “split hand”^7^ phenomenon in ALS.


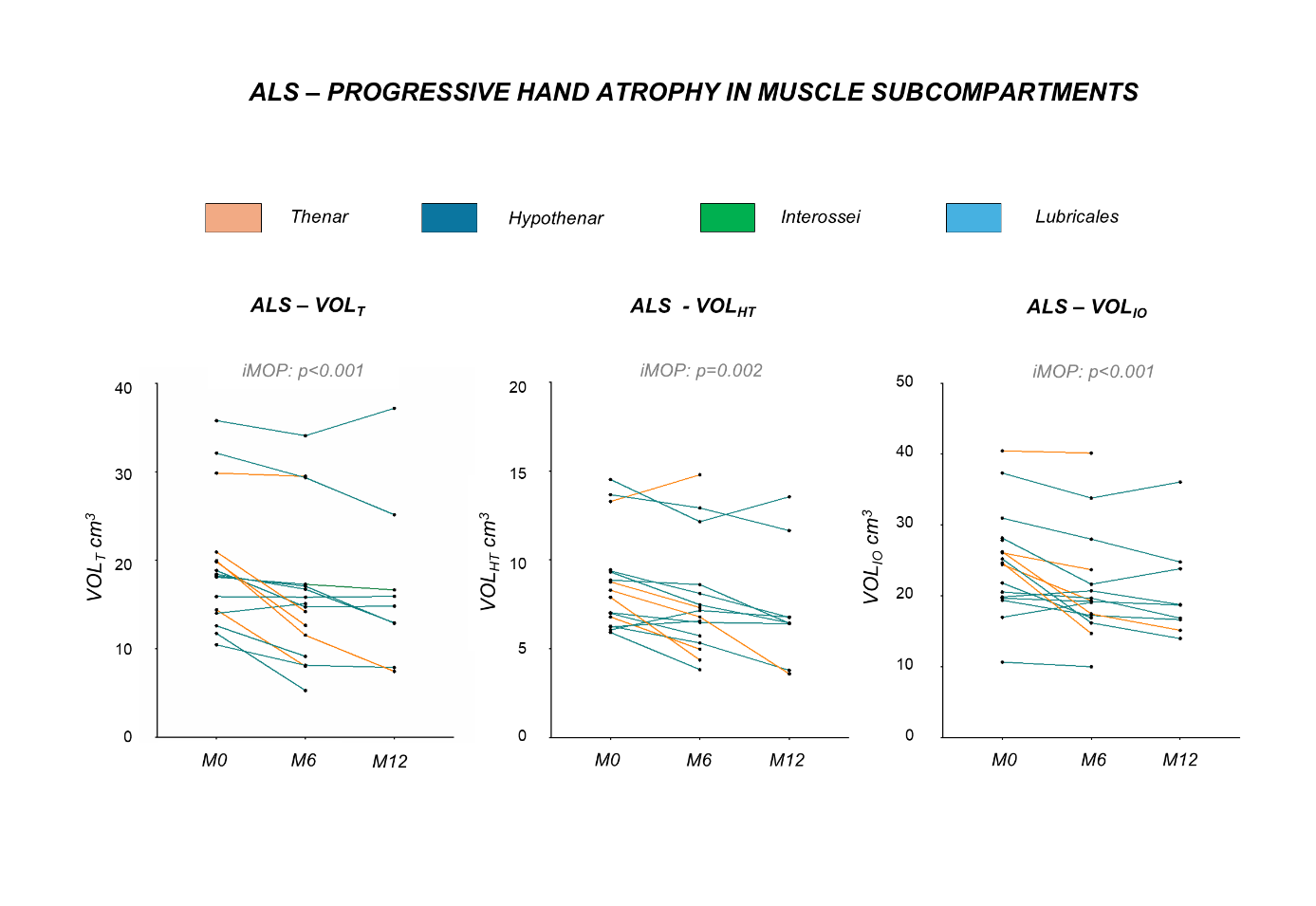


**Supplementary Figure 1**. Hand muscle subgroup volumetrics over time in thenar (VOL*_T_*, left), hypothenar (VOL*_HT_*, middle) and interossei muscles (VOL*_IO_*, right) in ALS patients. Data are shown as before-after plots of individual values at baseline (M0), 6 months (M6) and 12 months (M12). Volumes of all investigated hand muscles significantly decreased over time (VOL*_T_*: test statistic=-120, p<0.001; VOL*_HT_*: test statistic=-114, p=0.002; VOL*_IO_*: test statistic=-124, p<0.001).

Note: ALS: amyotrophic lateral sclerosis; M0: baseline; M6: 6-month follow up; M12: 12-month follow-up; VOL*_IO_*: and interossei muscle volume; VOL*_HAND_*: hand volume; VOL*_T_*, left), VOL*_HT_*: hypothenar muscle volume.


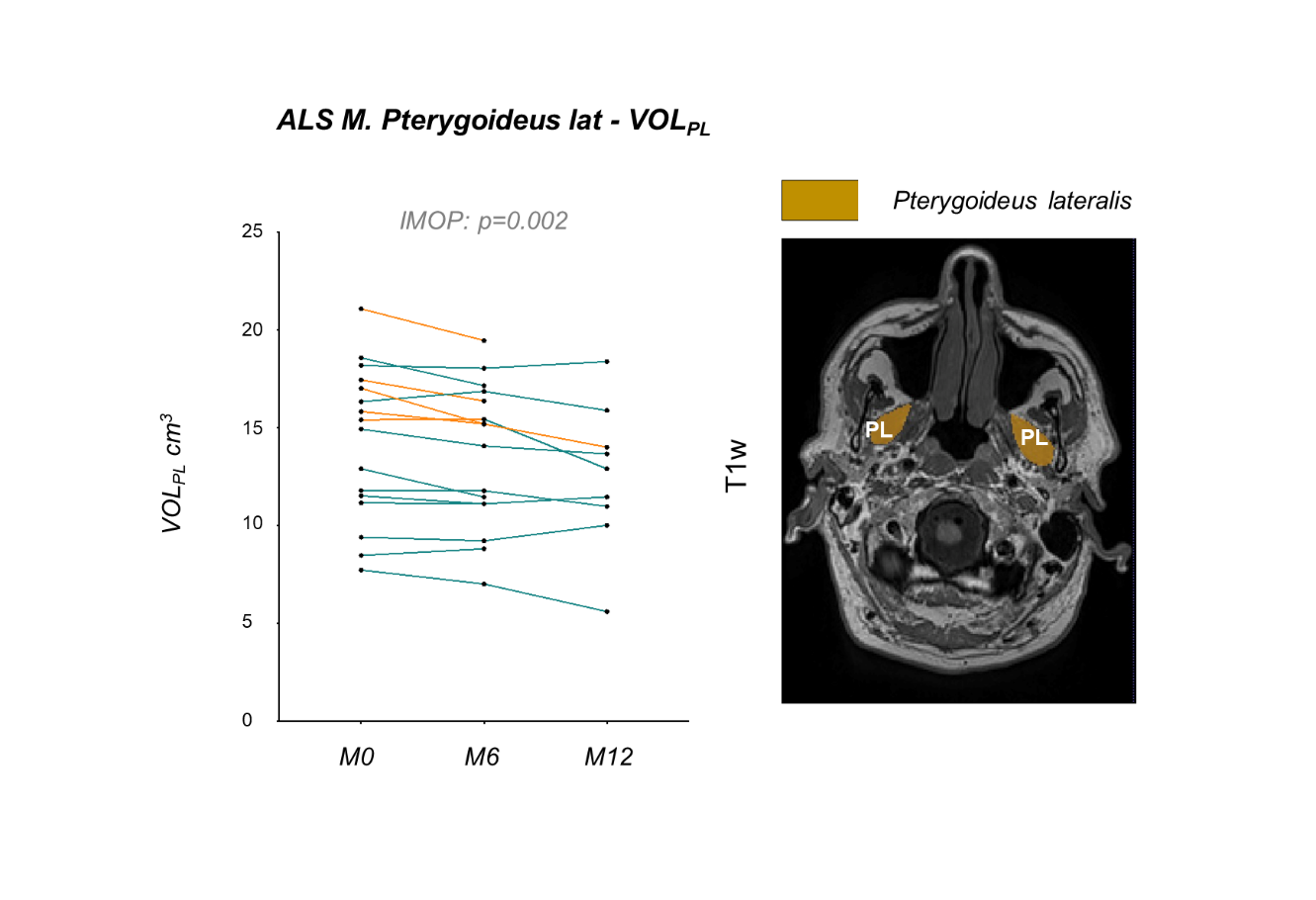


**Supplementary Figure 2.** Volume of the bilateral pterygoideus lateralis muscles (VOL*_PL_*, left) with corresponding T1w sample image of an ALS patient (right). Data are shown as before-after plots of individual values at baseline (M0), 6 months (M6) and 12 months (M12). A significant decline in muscle volume VOL*_PL_* was observed over the individual maximum observation period (iMOP; T(1,15)=3.66, p=0.002).

Note: VOL*_PL_*: muscle specific volume of pterygoideus lateralis muscles, iMOP: individual maximum observation period).

**Supplementary Table 4**. Change in quantitative MRI parameters between baseline vs. 6 months and baseline vs. individual maximum observation period (iMOP) in ALS patients.

|  | **baseline vs. 6 months** | | | | |  | **baseline vs. iMOP** | | | |
| --- | --- | --- | --- | --- | --- | --- | --- | --- | --- | --- |
| **Atrophy** | **Mean change** | **SD** | **SRM** |  | **Mean change** | | | **SD** | **SRM** |  |
| CSA*_CALF_* (cm²) | -15.06 | 17.63 | -0.85 |  | -22.64 | | | 20.89 | -1.08 |  |
| CSA*_THIGH_* (cm²) | -24.34 | 24.44 | -1.00 |  | -38.12 | | | 34.82 | -1.09 |  |
| VOL*_HAND_* (cm³) | -8.37 | 7.29 | -1.15 |  | -10.56 | | | 9.01 | -1.17 |  |
| VOL*_T_* (cm³) | -3.28 | 2.93 | -1.12 |  | -4.15 | | | 3.72 | -1.12 |  |
| VOL*_HT_* (cm³) | -1.04 | 1.28 | -0.81 |  | -1.64 | | | 1.59 | -1.03 |  |
| VOL*_IO_* (cm³) | -3.42 | 3.67 | -0.93 |  | -3.98 | | | 3.97 | -1.00 |  |
| VOL*_PL_* (cm³) | -0.60 | 0.73 | -0.83 |  | -0.96 | | | 1.05 | -0.92 |  |
|  |  |  |  |  |  | | |  |  |  |
| **Fat infiltration** | **Mean change** | **SD** | **SRM** |  | **Mean change** | | | **SD** | **SRM** |  |
| FF*_CALF_* (%) | 1.30 | 1.71 | 0.76 |  | 2.75 | | | 3.38 | 0.81 |  |
| FF*_THIGH_* (%) | 0.47 | 0.82 | 0.58 |  | 1.02 | | | 1.73 | 0.59 |  |
| FF*_HAND_* (%) | 1.59 | 2.79 | 0.57 |  | 1.97 | | | 3.26 | 0.61 |  |
| FF*_TONGUE_* (%) | 5.93 | 8.03 | 0.74 |  | 4.15 | | | 8.90 | 0.47 |  |
| fRMA*_CALF_* (cm^2^) | -15.61 | 17.59 | -0.89 |  | -24.08 | | | 21.80 | -1.10 |  |
| fRMA*_THIGH_* (cm^2^) | -24.12 | 23.98 | -1.01 |  | -37.92 | | | 34.22 | -1.11 |  |
| fRMA*_TONGUE_* (cm²) | -1.88 | 4.71 | -0.40 |  | -1.05 | | | 5.68 | -0.19 |  |
|  |  |  |  |  |  | | |  |  |  |
|  |  |  |  |  |  | | |  |  |  |
| **T_2m_** | **Mean change** | **SD** | **SRM** |  | **Mean change** | | | **SD** | **SRM** |  |
| T_2m_*_CALF_* (ms) | 1,58 | 1,56 | 1,02 |  | 2,27 | | | 3,07 | 0,74 |  |
| T_2m_*_THIGH_* (ms) | 0,82 | 1,34 | 0,61 |  | 0,87 | | | 1,45 | 0,60 |  |


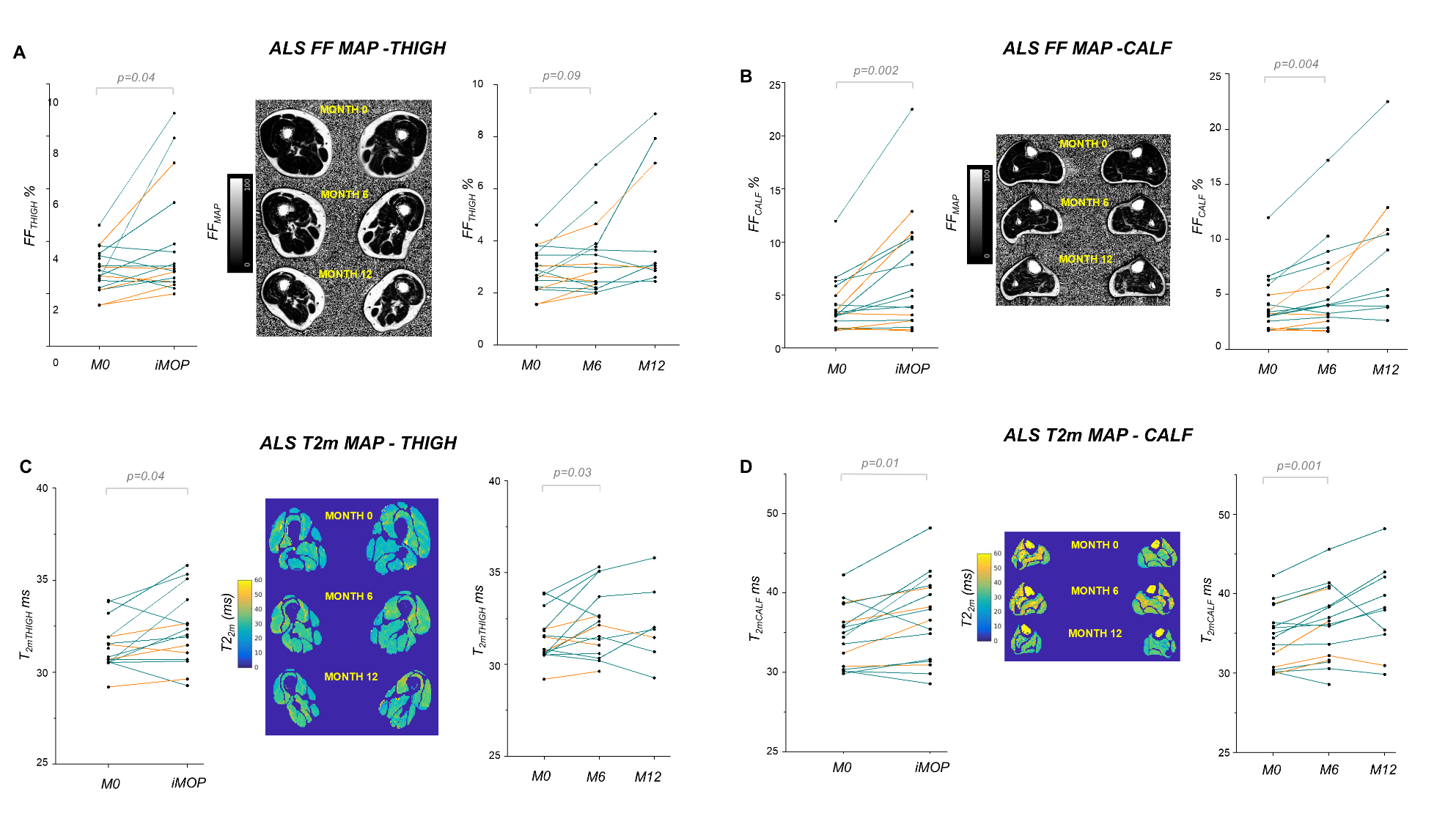


**Supplementary Figure 3. Fat fraction and water T2changes in ALS thigh and calf muscles**

**(A)** Overall muscle fat fraction at thigh level (FF*_THIGH_*; left panel) over time in ALS patients (fast progressors: orange; slow progressors: green). Left panel: mean values at baseline (M0) and individual maximum observation period (iMOP). Right panel: individual values at baseline (M0), 6 months (M6) and 12 months (M12)*.* FF*_THIGH_* shows a trend to increase over the iMOP in ALS patients (Bonferroni corrected p>0.003). Middle panel: Sample FF map axial images of thighs of an ALS patient at baseline (upper row), 6 months (middle row) and 12 months (lower row).

**(B)** Overall muscle fat fraction at calf level (FF*_CALF_*) over time in ALS patients. Left panel: mean values at baseline (M0) and individual maximum observation period (iMOP). Right panel: individual values at baseline (M0), 6 months (M6) and 12 months (M12). A significant increase in overall FF*_CALF_* over the iMOP (test statistic=123, p=0.002) was observed. Middle panel: Sample FF map axial images of calves of an ALS patient at baseline (upper row), 6 months (middle row) and 12 months (lower row). **(C)** Overall water T2 at thigh level (T_2m_*_THIGH_*) over time in ALS patients. Left panel: mean values at baseline (M0) and individual maximum observation period (iMOP). Right panel: individual values at baseline (M0), 6 months (M6) and 12 months (M12). No significant change in T_2m_*_THIGH_* was observed (Bonferroni corrected p>0.003). Middle panel: Sample T2 axial images of thighs of an ALS patient at baseline, 6 months and 12 months. **(D)** Overall water T2 at calf level (T_2m_*_CALF_*) over time in ALS patients. Left panel: mean values at baseline (M0) and individual maximum observation period (iMOP). Right panel: individual values at baseline (M0), 6 months (M6) and 12 months (M12). No significant change in T_2m_*_CALF_* was observed (Bonferroni corrected p>0.003). Middle panel: Sample T2 axial images of calves of an ALS patient at baseline, 6 months and 12 months.

Note: ALS: amyotrophic lateral sclerosis; FF: fat fraction; FF*_THIGH_*: overall fat fraction of thigh muscles. FF*_CALF_*: overall fat fraction of calf muscles. FF*_ANT-TMC_*: muscle specific fat fraction of anterior thigh muscle compartment; FF*_POSD-CMC_*_:_ muscle specific fat fraction of posterior deep calf muscle compartment; iMOP,:(individual maximum observation period); M0: baseline; M6: 6-month follow up; M12: 12-month follow-up; T_2m_*_CALF_*: overall water T2 at calf level; T_2m_*_THIGH_*: overall water T2 at thigh level.

**Supplementary Table 5**. Quantitative MRI parameters in ALS patients at baseline and 6-month follow-up. Data are presented as mean±SD or median (IQR) according to data distribution.

| **Atrophy** | **Baseline** | **6-month follow-up** | **p** | **95% CI** |
| --- | --- | --- | --- | --- |
| CSA*_CALF_* (cm²) | 112.80±33.17 | 100.69±28.92 | **0.004** | -24.46 – -5.67 |
| CSA*_THIGH_* (cm²) | 183.33±59.24 | 161.86±57.92 | **<0.001** | -39.61 – -11.78 |
| VOL*_HAND_* (cm³) | 57.04 (16.39) | 45.18 (14.82) | **<0.001** | -12.25 – -1.28 |
| VOL*_PL_* (cm³) | 14.24±3.92 | 13.64±3.64 | **0.002** | -0.99 – -0.21 |
| **Fat infiltration** | **Baseline** | **6-month follow-up** | **p** | **95% CI** |
| FF*_CALF_* (%) | 3.34 (2.07) | 3.99 (4.84) | **0.004** | 0.42–2.18 |
| FF*_THIGH_* (%) | 2.79 (1.13) | 2.96 (1.56) | 0.09 | 0.05–0.90 |
| FF*_HAND_* (%) | 2.22 (3.47) | 5.54 (4.10) | 0.07 | -0.18–3.36 |
| FF*_TONGUE_* (%) | 8.50± 3.42 | 14.10 (14.11) | 0.02 | 1.08–10.78 |
| fRMA*_CALF_* (cm^2^) | 108.48±32.67 | 95.90±29.31 | **0.003** | 6.24–2.50 |
| fRMA*_THIGH_* (cm^2^) | 178.12±57.59 | 156.81±56.83 | **<0.001** | 11.79–36.45 |
| fRMA*_TONGUE_* (cm^2^) | 25.94±4.97 | 23.47±5.28 | 0.09 | -0.97–4.73 |
|  |  |  |  |  |
| **T_2m_** | **Baseline** | **6-month follow-up** | **p** | **95% CI** |
| T_2m_*_CALF_* (ms) | 34.61±3.85 | 36.19±4.65 | **0.001** | -2.42 – -0.75 |
| T_2m_*_THIGH_* (ms) | 31.43±1.34 | 32.25±1.85 | **0.03** | -1.56 – -0.08 |

**Supplementary Table 6**. Correlations of longitudinal changes of quantitative MRI parameters with changes in functional rating scales and muscle strength assessments over time in ALS patients.

| **Functional rating scales** | **Correlation coefficient** | **p** | **95%CI** |  |
| --- | --- | --- | --- | --- |
| ALSFRS*_TOTAL_* × fRMA*_THIGH_* | 0.52 | 0.03 | 0.05 – 0.80 |  |
| ALSFRS*_TOTAL_* × fRMA*_CALF_* | 0.68, | 0.004 | 0.28 – 0.88 |  |
| ALSFRS*_LL-SS_* × fRMA*_THIGH_* | 0.39 | 0.12 | 0.12 – 0.37 |  |
| ALSFRS*_LL-SS_* × fRMA*_CALF_* | 0.16 | 0.55 | -0.36 – 0.61 |  |
| ALSFRS*_HAND-SS_* × VOL*_HAND_* | 0.53 | 0.03 | 0.05 – 0.81 |  |
| ALSFRS*_BULBAR-SS_* × fRMA*_TONGUE_* | 0.78 | 0.003 | 0.38 – 0.93 |  |
| ALSFRS*_BULBAR-SS_* × VOL*_PL_* | 0.45 | 0.04 | -0.07 – 0.78 |  |
|  |  |  |  |  |
| **Muscle strength assessments** | **Correlation coefficient** | **p** | **95%CI** |  |
| Knee extension × fRMA*_ANT-TMC_* | 0.77 | 0.001 | 0.46 – 0.91 |  |
| Plantar flexion × fRMA*_TRICEPSSUR_* | 0.78 | <0.001 | 0.47 – 0.92 |  |
| Grip × VOL*_HAND_* | 0.71 | 0.004 | 0.29 – 0.90 |  |
| IOPI × fRMA*_TONGUE_* | 0.27 | 0.48 | -0.67–1.30 |  |
| Jaw opening × VOL*_PL_* | 0.15 | 0.59 | -0.39 – 0.61 |  |

***Muscle fat fraction (FF) increases over time***

FF*_THIGH_* showed a trend to increase over the iMOP (Bonferroni corrected p>0.003; **Table 1, Supplementary Figure 2**), while FF*_CALF_* increased significantly over time (rs=0.84; test statistic=123, p=0.002; **Table 1**, **Supplementary Figure 2)**. Similarly, a significant increase in overall FF*_HAND_* was observed over iMOP (rs=0.46; test statistic=105.0, p<0.001, **Table 1)**. At head-neck level, no significant change in FF*_TONGUE_* was observed over iMOP (partial η²=0.11; T(1,13)=1.22, p=0.25; **Table 1**).

***Progressive loss of fRMA and muscle volume correlate with disease severity***

***Head and neck***

At head-neck level, the relative decrease of overall tongue fRMA*_TONGUE_* significantly correlated with the decrease of the subscale ALSFRS*_BULBAR-SS_* over time (rho=0.78, p=0.003, **Supplementary Table 6**).

The correlation of the relative volume decrease of the bilateral pterygoideus lateralis muscles with the decrease of the subscale ALSFRS*_BULBAR-SS_* over the iMOP was not significant (Bonferroni corrected p>0.004, **Supplementary Table 6**). No significant correlations between quantitative MRI parameters and myometry assessments were observed at head-neck level.

## Exploratory comparison of MRI parameter correlations with disease severity in fast vs. slow progressors

We performed exploratory analyses comparing the correlations of progressive MRI parameter changes with decrease of muscle strength and functional rating scale scores in fast (n=6) and slow (n=11) disesase progressors. For the correlation of the relative decrease of fRMA*_ANT-TMC_* of the anterior thigh muscle compartment with the relative decrease of knee extension strength over time (r=0.77, p<0.001; **Supplementary Table 7**, **Figure 1**), there was no significant difference (z=0.04, p=0.97) between fast progressors (r=0.77, p=0.07) and slow progressors (r=0.76, p=0.01). For the correlation of the relative decrease of fRMA*_TRICEPSSUR_* with the relative decrease of plantar flexion strength over the iMOP (r=0.78, p<0.001; **Supplementary Table 7, Figure 1**), there was again no difference between fast progressors (r=0.86, p=0.06) and slow progressors (r=0.86, p<0.001; z=-0.03, p=0.98).

The correlation of the ALSFRS*_TOTAL_* score with the relative decrease of fRMA at calf level was stronger in fast progressors (r=0.95, p=0.01) than slow progressors (r=0.001, p=0.99) at calf level (p=0.01, z=2.70). The correlation of the relative decrease of VOL*_HAND_* with the corresponding relative decrease of hand grip strength did not differ between fast (r=0.52, p=0.48) and slow progressors (r=0.80, p=0.005; z=-0.65, p=0.52, **Supplementary Table 7**). For the correlation of overall tongue fRMA_TONGUE_ with the decrease of the subscale ALSFRS*_BULBAR-SS_* a comparison of correlations between fast progressors and slow progressors could not be performed due to loss of follow-up of 3 fast progressors.

**Supplementary Table 7.** Correlations of longitudinal changes of quantitative MRI parameters with changes in functional rating scales and muscle strength assessments over time in fast and slow progessing ALS patients.

|  | **Functional rating scales** | **Correlation coefficient** | **p** | **95%CI** |  |
| --- | --- | --- | --- | --- | --- |
| ALSFRS*_TOTAL_* × fRMA*_THIGH_* | fast progressors | 0.97 | 0.001 | 0.75 – 1.00 |  |
|  | slow progressors | -0.30 | 0.37 | 0.76 – 0.37 |  |
| ALSFRS*_TOTAL_* × fRMA*_CALF_* | fast progressors | 0.95 | 0.01 | 0.43 – 1.00 |  |
|  | slow progressors | 0.001 | 0.99 | 0.60 – 0.60 |  |
| ALSFRS*_LL-SS_* × fRMA*_THIGH_* | fast progressors | 0.87 | 0.02 | 0.19 – 0.99 |  |
|  | slow progressors | -0.12 | 0.74 | 0.67 – 0.52 |  |
| ALSFRS*_LL-SS_* × fRMA*_CALF_* | fast progressors | -0.16 | 0.79 | 0.91 – 0.84 |  |
|  | slow progressors | 0.09 | 0.80 | 0.54 – 0.65 |  |
| ALSFRS*_HAND-SS_* × VOL*_HAND_* | fast progressors | 0.27 | 0.66 | -0.80 – 0.93 |  |
|  | slow progressors | 0.64 | 0.03 | 0.07 – 0.90 |  |
| ALSFRS*_BULBAR-SS_* × fRMA*_TONGUE_* | fast progressors | n.a. | n.a. | n.a. |  |
|  | slow progressors | n.a. | n.a. | n.a. |  |
| ALSFRS*_BULBAR-SS_* × VOL*_PL_* | fast progressors | 0.44 | 0.46 | -0.73 – 0.95 |  |
|  | slow progressors | 0.37 | 0.35 | -0.35 – 0.79 |  |
|  |  |  |  |  |  |
|  | **Muscle strength assessments** | **Correlation coefficient** | **p** | **95%CI** |  |
| Knee extension × fRMA*_ANT-TMC_* | fast progressors | 0.77 | 0.07 | -0.11 – 0.97 |  |
|  | slow progressors | 0.76 | 0.01 | 0.29 – 0.93 |  |
| Plantar flexion × fRMA*_TRICEPSSUR_* | fast progressors | 0.86 | 0.06 | -0.09 – 0.99 |  |
|  | slow progressors | 0.86 | <0.001 | 0.53 – 0.96 |  |
| Grip × VOL*_HAND_* | fast progressors | 0.52 | 0.48 | -0.88 – 0.99 |  |
|  | slow progressors | 0.80 | 0.005 | 0.35 – 0.95 |  |
| IOPI × fRMA*_TONGUE_* | fast progressors | n.a. | n.a. | n.a. |  |
|  | slow progressors | 0.24 | 0.57 | -0.92–1.53 |  |
| Jaw opening × VOL*_PL_* | fast progressors | 0.29 | 0.64 | -0.80 – 0.93 |  |
|  | slow progressors | 0.16 | 0.65 | -0.52 – 0.72 |  |

**Exploratory analysis of the relation between progressive MRI parameter changes and the respiratory subscale of ALSFRS-R**

We performed an exploratory analysis of the relationship between the respiratory subscale of the ALSFRS-R (ALSFRS-R*_RESP_*) and longitudinal MRI parameter changes.

There was a significant correlation between ALSFRS-R*_RESP_* decline and the relative decrease in fRMA*_CALF_* (r=0.54, p=0.03) and fRMA*_TONGUE_* (r=0.57, p=0.04). However, correlations with fRMA*_THIGH_* (r=-0.41, p=0.11), VOL*_HAND_* (r=0.12, p=0.65) and VOL*_PL_* (rho=0.36, p=0.17) were not significant. While this study did not specifically investigate respiratory muscles via MRI protocol, these findings emphasize the potential relationship between respiratory function and muscle atrophy in ALS. Future studies should explore these relations in larger samples and include quantitative respiratory parameters, such as forced vital capacity (FVC), tidal volume, or diaphragm imaging, to better understand their contribution to disease progression.

**References**

1. Brooks BR, Miller RG, Swash M, Munsat TL, World Federation of Neurology Research Group on Motor Neuron Diseases. El Escorial revisited: revised criteria for the diagnosis of amyotrophic lateral sclerosis. *Amyotroph Lateral Scler Mot Neuron Disord Off Publ World Fed Neurol Res Group Mot Neuron Dis*. 2000;1(5):293-299.

2. Klickovic U, Zampedri L, Sinclair CDJ, et al. Skeletal muscle MRI differentiates SBMA and ALS and correlates with disease severity. *Neurology*. 2019;93(9):e895-e907. doi:10.1212/WNL.0000000000008009

3. Cedarbaum JM, Stambler N, Malta E, et al. The ALSFRS-R: a revised ALS functional rating scale that incorporates assessments of respiratory function. *J Neurol Sci*. 1999;169(1-2):13-21. doi:10.1016/S0022-510X(99)00210-5

4. Sipers WMWH, Verdijk LB, Sipers SJE, Schols JMGA, van Loon LJC. The Martin Vigorimeter Represents a Reliable and More Practical Tool Than the Jamar Dynamometer to Assess Handgrip Strength in the Geriatric Patient. *J Am Med Dir Assoc*. 2016;17(5):466.e1-466.e7. doi:10.1016/j.jamda.2016.02.026

5. Adams V, Mathisen B, Baines S, Lazarus C, Callister R. A Systematic Review and Meta-analysis of Measurements of Tongue and Hand Strength and Endurance Using the Iowa Oral Performance Instrument (IOPI). *Dysphagia*. 2013;28(3):350-369. doi:10.1007/s00455-013-9451-3

6. Yushkevich PA, Piven J, Hazlett HC, et al. User-guided 3D active contour segmentation of anatomical structures: Significantly improved efficiency and reliability. *NeuroImage*. 2006;31(3):1116-1128. doi:10.1016/j.neuroimage.2006.01.015

7. Eisen A, Kuwabara S. The split hand syndrome in amyotrophic lateral sclerosis. *J Neurol Neurosurg Psychiatry*. 2012;83(4):399-403. doi:10.1136/jnnp-2011-301456
